# Validation of self-report of uterine fibroid diagnosis using a transvaginal ultrasound scan

**DOI:** 10.1038/s41598-023-36313-y

**Published:** 2023-06-05

**Authors:** Clement A. Adebamowo, Imran O. Morhason-Bello, The ACCME Research Group as part of the H3Africa Consortium, Ayotunde O. Famooto, Richard Offiong, Olayinka Olaniyan, Kayode Obende, Amos Adebayo, Sanni Ologun, Bunmi Alabi, Peter Achara, Sally N. Adebamowo

**Affiliations:** 1 Department of Epidemiology and Public Health, School of Medicine, University of Maryland, 660 West Redwood Street, Baltimore, MD 21201 USA; 2 Greenebaum Comprehensive Cancer Center, School of Medicine, University of Maryland, Baltimore, MD USA; 3 Center for Bioethics and Research, Ibadan, Nigeria; 4 Institute of Human Virology Nigeria, Abuja, Nigeria; 5 Department of Obstetrics and Gynecology, Faculty of Clinical Sciences, and the Institute of Advanced Medical Research and Training College of Medicine University of Ibadan, Ibadan, Nigeria; 6 University of Abuja Teaching Hospital, Gwagwalada, Abuja, Nigeria; 7 National Hospital Abuja, Abuja, Nigeria; 8 Garki Hospital Abuja, Abuja, Nigeria; 9 Asokoro District Hospital, Abuja, Nigeria; 10 Kubwa General Hospital, Abuja, Nigeria; 11 Wuse General Hospital, Abuja, Nigeria; 12 Federal Medical Center, Keffi, Nigeria

**Keywords:** Urogenital diseases, Signs and symptoms

## Abstract

Self-report of uterine fibroids (UF) has been used for epidemiologic research in different environments. Given the dearth of studies on the epidemiology of UF in Sub-Saharan Africa (SSA), it is valuable to evaluate its performance as a potential tool for much needed research on this common neoplasm in SSA women. We conducted a cross-sectional study of self-report of UF compared with transvaginal ultrasound diagnosis (TVUS) among 486 women who are members of the African Collaborative Center for Microbiome and Genomics Research (ACCME) Study Cohort in central Nigeria. We used log-binomial regression models to compute the classification, sensitivity, specificity, and predictive values of self-report compared to TVUS, adjusted for significant covariates. The prevalence of UF on TVUS was 45.1% (219/486) compared to 5.4% (26/486) based on self-report of abdominal ultrasound scan and 7.2% (35/486) based on report of healthcare practitioner’s diagnosis. Self-report correctly classified 39.5% of the women compared to TVUS in multivariable adjusted models. The multivariable adjusted sensitivity of self-report of healthcare worker diagnosis was 38.8%, specificity was 74.5%, positive predictive value (PPV) was 55.6%, and negative predictive value (NPV) was 59.8%. For self-reported abdominal ultrasound diagnosis, the multivariable adjusted sensitivity was 40.6%, specificity was 75.3%, PPV was 57.4%, and NPV was 60.6%. Self-report significantly underestimates the prevalence of UF and is not accurate enough for epidemiological research on UF. Future studies of UF should use population-based designs and more accurate diagnostic tools such as TVUS.

## Introduction

Uterine fibroid or uterine leiomyoma (UF) is the most common solid and symptomatic neoplasm in women globally and in Sub-Saharan Africa (SSA)^1,2^. There is a dearth of data on its prevalence, but studies from developed countries show that the cumulative incidence of UF by the age of 50 years is 70–80%^1,3^. The incidence and prevalence of UF varies significantly by race, and all studies with race data report a higher incidence and prevalence in women of African ancestry^3–5^. Though benign and frequently asymptomatic, UF is associated with significant morbidities, including menorrhagia and other menstrual abnormalities, anemia, pelvic pain, infertility, pregnancy complications, and occasional mortality^6–8^. Globally, the total direct and indirect costs of UF after diagnosis or from surgical care ranged from US$11,717 to $25,023 per patient per year^9^.

There have been very few well-conducted studies of the epidemiology of UF in SSA. In a recent scoping review of several databases^10^, we identified only nine epidemiologic studies of UF in SSA^11–19^. Of these, two were hospital-based case–control studies, while the others used different study populations, sampling methods, study designs, and methods of analyses^10^. These studies mostly reported on women who were diagnosed at clinics, pathology, or ultrasound departments^10^. However, data from developed countries estimate that UF is clinically apparent in only 25% of women of reproductive age and leads to clinical presentation in only 25% of these clinically apparent cases^1^. Hospital-based studies are therefore likely to significantly underestimate the burden of UF in the population and are subject to selection bias.

Self-report of clinical conditions is a commonly used tool in epidemiological research. Almost 90% of urbanized Nigerian women and 97% of those who have received at least 12 years of education receive ante-natal care during which abdominal ultrasounds are typically performed^20^. Report of being informed about the presence of UF by healthcare professionals following clinical examination or from abdominal ultrasound scan may be considered valuable tools for epidemiological studies of UF in this and similar populations. However, because most UF cases are asymptomatic and the symptoms of UF are nonspecific, self-report of UF based on women’s recollection of clinicians’ or abdominal ultrasonographic diagnosis is also likely to lead to significant misclassification^21^.

In this study, we compare self-report of healthcare workers’ and abdominal ultrasound diagnosis of UF with transvaginal ultrasound diagnosis of UF among women enrolled in the African Collaborative Center for Microbiome and Genomics Research (ACCME) prospective cohort in Abuja, Central Nigeria.

## Methods

The ACCME cohort study profile and the characteristics of the study participants have been previously described in detail^22^. We enrolled 11,700 women who were at least 18 years old at enrollment, HIV negative, and had a history of penetrative vaginal intercourse but no previous history of cervical abnormalities, cervical cancer, or hysterectomy into the ACCME cohort from 2014 to 2016. Trained research nurses administered validated questionnaires to collect demographic, lifestyle, obstetric and gynecologic history, medical history, sexual behavior and practices, diet, and physical activities data.

In 2020, for the UF study, we developed and internally validated a supplemental questionnaire among 20 ACCME participants, who were not included in the rest of the UF study. The UF related questions in the questionnaire were:

- Has a doctor ever told you that you have uterine fibroid and you have not received any treatment for it?
- Has a doctor ever told you that you do not have uterine fibroid?
- Have you ever been told that you have a uterine fibroid on abdominal ultrasound scan and have not received treatment for it?
- Have you ever been told that you do not have uterine fibroids on abdominal ultrasound scan?
- Do you have any symptoms—excessive bleeding during menstrual periods, severe pelvic/abdominal pain, inability to conceive, abdominal swelling—that have been attributed to uterine fibroids?

We identified 486 participants from the ACCME cohort using random numbers generated using Stata 17® software. All those invited responded and consented to participate in this study. The participants’ epidemiological data were retrieved from the ACCME Cohort baseline dataset. The variables retrieved included age in years, years of education completed (elementary, completed high school, post-high school with no university degree, completed university), marital status (married, single, separated/divorced/widowed), occupation, smoking experience (yes vs. no), alcohol use (yes vs. no), age at menarche, number of pregnancies, which was categorized into 0, 1–2, 3–5, > 5), spontaneous or medical abortions, ever used contraceptive (yes vs. no), menopausal status (premenopausal vs. postmenopausal), sexual history, practices and hygiene, breastfeeding experience of more than one month (yes vs. no) and genital symptoms. To compute socio-economic status (SES), we calculated the ‘wealth index’ using the following variables: house ownership and type of house owned (e.g., home, apartment, house or duplex); source of drinking water (e.g., from outside, well, borehole, piped or bottled); type of cooking fuel; use of separate room for cooking; type of toilet; and ownership of household goods including car and refrigerator. We used principal component analysis (PCA) with varimax rotation to compute factor scores based on the sum of responses to these variables weighted by their factor loading. We used the first component in the PCA that explained 35% of the variation in the data to generate a wealth index (39). The wealth index variable was used to classify participants into low (lowest 50% of the score distribution), middle (middle 30%) and high (highest 20%) SES categories.

Participants were offered a transvaginal ultrasound scan (TVUS) performed by clinical radiologists whose training was certified by the West African College of Surgeons. UF identification was based on the Muram criteria and included all relatively spherical, echogenically distinct masses in the myometrium that were more than 0.5 cm in diameter^23^. Participants with UF on TVUS were referred to the gynecologic clinic for standard of care treatment.

Using TVUS as the gold standard, we conducted univariate analyses of the associations between individual predictors and the classification (agreement), sensitivities, specificities, and predictive values of (a) being told about the diagnosis of UF by doctors and (b) being told about the presence of UF after abdominal ultrasonography^24^. We used the benchmark scale of Landis and Koch, which categorized agreement to < 0.00 Poor, 0.00–0.20 Slight 0.21–0.40 Fair, 0.41–0.60 Moderate, 0.61–0.80 Substantial, and 0.81–1.00 Almost perfect, to interpret the agreement statistic^25^. We included variables with p-values < 0.10 on bivariable analyses in multivariable log-binomial regression models to compute adjusted sensitivities and specificities. We analyzed all data using Stata 17® (College Station, Texas 77845 USA).

### Patient and public involvement

Patients and the public were not involved in the design and analysis of this project. The study is population based and thus did not enrol individuals who presented as patients in a medical institution. The findings on TVUS were shared with participants who were counselled on accessing the standard of care for UF in Nigeria. The idea and design of the study was based on clinical experience with patients, but those patients were not involved in the recruitment or conduct of this study.

The study protocol was reviewed and approved by the National Health Research Ethics Committee of Nigeria (NHREC Approval Number NHREC/01/01/2007–29/11/2016) and an IRB authorization agreement with the University of Maryland School of Medicine. All research methods were performed in accordance with the relevant clinical and diagnostic guidelines in Nigeria. ACCME study participants receive the project newsletter quarterly, and the results of this study are shared with them through this mechanism. The ACCME Project has an active website and social media that is used to engage study participants and disseminate the results of this study.

### Patient consent for publication

Informed consent was obtained from all study participants.

### Ethical approval

Ethical approval was obtained from the Nigerian National Health Research Ethics Committee and the University of Maryland School of Medicine IRB.

## Results

The prevalence of UF on TVUS in this study population was 45.1% (219/486). Only 5.4% (26/486) of the participants reported being informed that they had UF after abdominal ultrasound scan, while 35 women (7.2%) reported that they had been informed that they had UF by a healthcare practitioner. The mean (SD) age of the participants was 37.1 (9.2) years, and this was not significantly different between women with UF on TVUS (mean (SD) = 37.0 (9.0) years) and those without UF on TVUS (mean (SD) = 37.1 (9.4) years) (Table 1). The age at menarche, age at sexual debut, SES, employment status, religion, marital status, history of use of any type of contraceptive, menopausal status, number of children, history of miscarriages or stillbirths or number of vaginal intercourse partners were not significantly different between women with UF and women without UF on TVUS (Table 1).

**Table 1:** Characteristics of women with or without uterine fibroids by transvaginal ultrasound scan, Nigeria, 2022.

	TVS result						P-value
	Negative (N = 267)		Positive (N = 219)		Total (N = 486)		
	Mean (SD)	N (%)	Mean (SD)	N (%)	Mean (SD)	N (%)	
Age (years)	37.1 (9.4)		37.0 (9.0)		37.1 (9.2)		0.83
Age at menarche (Years)	14.3 (2.0)		14.3 (1.9)		14.3 (1.9)		0.99
Sexual debut age (Years)	19.8 (3.9)		20.1 (3.9)		19.9 (3.9)		0.38
Age group							
18–29		64 (24.0%)		50 (22.8%)		114 (23.5%)	0.48
30–39		90 (33.7%)		85 (38.8%)		175 (36.0%)	
40–49		89 (33.3%)		60 (27.4%)		149 (30.7%)	
50 and above		24 (9.0%)		22 (10.1%)		46 (9.5%)	
Missing		0 (0.0%)		2 (0.9%)		2 (0.4%)	
Socio-economic status							
Low		126 (25.9%)		108 (22.2%)		234 (48.2%)	0.31
Middle		89 (18.3%)		68 (14.0%)		157 (32.3%)	
High		52 (10.7%)		43 (8.9%)		95 (19.6%)	
Education							
No formal schooling		25 (9.4%)		22 (10.0%)		47 (9.7%)	0.03
1–6 years of schooling		46 (17.2%)		20 (9.1%)		66 (13.6%)	
7–12 years of schooling		74 (27.7%)		54 (24.7%)		128 (26.3%)	
University		122 (45.7%)		123 (56.2%)		245 (50.4%)	
Occupation							
Unemployed		42 (15.7%)		40 (18.3%)		82 (16.9%)	0.46
Employed		225 (84.3%)		179 (81.7%)		404 (83.1%)	
Religion							
Islam		55 (20.6%)		51 (23.3%)		106 (21.8%)	0.48
Christian		212 (79.4%)		168 (76.7%)		380 (78.2%)	
Marital status							
Unmarried		39 (14.6%)		47 (21.5%)		86 (17.7%)	0.05
Married		228 (85.4%)		172 (78.5%)		400 (82.3%)	
Contraceptive use							
No		130 (48.7%)		122 (55.7%)		252 (51.9%)	0.12
Yes		137 (51.3%)		97 (44.3%)		234 (48.1%)	
Age at menarche							
8–14		148 (55.4%)		125 (57.1%)		273 (56.2%)	0.88
15–19		112 (41.9%)		88 (40.2%)		200 (41.2%)	
20–24		6 (2.2%)		4 (1.8%)		10 (2.1%)	
Missing		1 (0.4%)		2 (0.9%)		3 (0.6%)	
Menopause							
No		200 (74.9%)		178 (81.3%)		378 (77.8%)	0.06
Yes		54 (20.2%)		30 (13.7%)		84 (17.3%)	
Not sure		13 (4.9%)		11 (5.0%)		24 (4.9%)	
Have you ever been pregnant							
No		14 (5.2%)		27 (12.3%)		41 (8.4%)	0.01
Yes		253 (94.8%)		192 (87.7%)		445 (91.6%)	
Sexual debut age							
Less than 15		10 (3.7%)		11 (5.0%)		21 (4.3%)	0.58
15–19		124 (46.4%)		90 (41.1%)		214 (44.0%)	
20–24		89 (33.3%)		82 (37.4%)		171 (35.2%)	
25 and above		43 (16.1%)		32 (14.6%)		75 (15.4%)	
Don’t know		1 (0.4%)		4 (1.8%)		5 (1.0%)	
Parity status							
Nulliparous		28 (10.5%)		43 (19.6%)		71 (14.6%)	< 0.01*
Parous		239 (89.5%)		176 (80.4%)		415 (85.4%)	
Number of children							
No child		28 (10.5%)		43 (19.6%)		71 (14.6%)	0.67
1–3		128 (47.9%)		98 (44.8%)		226 (46.5%)	
4–10		111 (41.6%)		78 (35.6%)		189 (38.9%)	
Miscarriage							
No		244 (91.4%)		203 (92.7%)		447 (92.0%)	0.60
Yes		23 (8.6%)		16 (7.3%)		39 (8.0%)	
Stillbirth							
No		254 (95.1%)		213 (97.3%)		467 (96.1%)	0.23
Yes		13 (4.9%)		6 (2.7%)		19 (3.9%)	
Abortion							
No		177 (66.3%)		166 (75.8%)		343 (70.6%)	0.02
Yes		90 (33.7%)		53 (24.2%)		143 (29.4%)	
Number of vaginal partners							
1		137 (51.3%)		103 (47.0%)		240 (49.4%)	0.17
2		54 (20.2%)		37 (16.9%)		91 (18.7)	
3 and above		72 (27.0%)		74 (33.8%)		146 (30.0%)	
Don’t know		4 (1.5%)		5 (2.3%)		9 (1.9%)	

Some 19.6% of the women with UF on TVUS (43/219) were nulliparous compared to 10.5% (28/267) of the women without UF on TVUS (*p*-value < 0.001). More women with UF on TVUS had never been pregnant (12.3%, 27/219) than women without UF (5.2%, 14/267) (*p*-value = 0.01). Ninety (33.7%) of the women without UF on TVUS terminated their pregnancies compared to 53 (24.2%) of women with UF. A greater proportion of the women without UF on TVUS (17.2%, 46/267) had only 1 to 6 years of schooling compared with women with UF on TVUS (9.1%, 20/219), and this was significantly different from other levels of education among the study population (*p-*value = 0.03). The marital status of women without UF was marginally significantly different from that of women with UF, with 14.6% (29/267) of the former unmarried compared with 21.5% (47/219) of the latter (*p*-value = 0.05).

The healthcare workers correctly classified participants in 56.4% (moderate) of instances, and this became 39.5% (fair) in multivariable models adjusted for age, menopausal status, parity, and history of abortion (Table 2). Comparing healthcare workers’ reports of the presence of UF with TVUS, the unadjusted sensitivity was 9.6%, but this increased to 38.8% in multivariable models (Table 2). The unadjusted specificity for healthcare workers’ reports was 94.8%, while the multivariable adjusted specificity was 74.5%, the positive predictive value (PPV) was 55.6%, and the negative predictive value (NPV) was 59.8%.

**Table 2:** Univariate and multivariable models of sensitivity, specificity, predictive values of report of health professionals’ and abdominal ultrasound scans’ diagnosis of uterine fibroid compared to TVUS diagnosis of uterine fibroid, Abuja, Nigeria 2022.

	TVUS result		Univariable models					Multivariable models*				
	Negative (N = 267)	Positive (N = 219)	Sensitivity (%)	Specificity (%)	Correct classification (Probabilistic benchmark interval)	Positive Predictive Value (PPV) (%)	Negative Predictive Value (NPV) (%)	Sensitivity (%)	Specificity (%)	Correct classification (Probabilistic benchmark interval)	Positive Predictive Value (PPV) (%)	Negative Predictive Value (NPV) (%)
	N (%)	N (%)										
Healthcare provider report on uterine fibroid												
No	253 (94.8)	198 (90.4)	9.6	94.8	56.4% (0.40–0.60%)	60.0	56.1	38.8	74.5	39.5% (0.20–0.40%)	55.6	59.8
Yes	14 (5.2)	21 (9.6)										
Abdominal ultrasound report on uterine fibroid												
No	258 (96.6)	202 (92.2)	7.8	96.6	56.7% (0.40–0.60%)	65.4	56.1	40.6	75.3	39.5% (0.20–0.40%)	57.4	60.6
Yes	9 (3.4)	17 (7.8)										

*The models were adjusted for age, menopausal status, parity and history of abortion.

Regarding history of receiving a diagnosis of UF after abdominal ultrasound scan, this report correctly classified participants in 56.7% (moderate), but this fell to 39.5% (fair) after adjustment for covariates. The unadjusted sensitivity was 7.8%, while the multivariable adjusted sensitivity was 40.6%. The unadjusted specificity was 96.6%, and it was 75.3% after adjustment for age, menopausal status, parity, and history of abortion. The unadjusted PPV of reporting the presence of UF on abdominal USS was 65.4%, the multivariable adjusted PPV was 57.4%, the unadjusted NPV was 56.1%, and the multivariable adjusted NPV was 60.6%.

## Discussion

In this study, we found fair to moderate agreement when comparing women’s reports of being told by healthcare workers about having UF or their reports of the presence of UF during abdominal ultrasound scans with TVUS diagnosis of UF. The self-report methods correctly classified a history of UF in less than 60% of cases, and the agreement between these methods and the gold standard fell below 40% after adjusting for significant covariates. The specificities of being told about UF diagnosis by healthcare workers or reporting same after abdominal ultrasound scans were high, but the sensitivities and predictive values were low and fell when adjusted for covariates. This suggests that the performance characteristics of self-report worsened with increasing age and parity, among postmenopausal women, and in women with a positive history of abortion.

Uterine fibroids are the most common symptomatic neoplasms of women globally, with significantly higher incidence, prevalence, and severity among women of African ancestry. However, there has been very little research on them, particularly among women in SSA, where they are associated with significant morbidities and some mortality which often arises as a complication of severe anaemia or surgical management. We recently reviewed published epidemiological studies of the incidence, prevalence, and risk factors for UF in indigenous African women, and we found that few studies had reliable information on the prevalence and risk factors for UF^11,13,15,17,19^. The studies were also generally flawed by choice of study populations, study designs, methods of diagnosis, sampling techniques and consideration of covariates^10^.

A potential method of conducting cost-effective, large-scale, epidemiological studies of UF is by use of self-report of symptoms, being told by healthcare worker about presence or absence of UF, or being told about diagnosis of UF after abdominal ultrasound scans. These methods have been used for UF research in other populations, but they are prone to misclassification^21,26^. The symptoms of UF are nonspecific; only ~ 25% of cases are symptomatic, and of these, only 25% present for clinical care in developed countries^1^. The proportion of women who are aware of the presence of UF and those presenting for care in low- and middle-income countries (LMICs) is likely to be much lower given low levels of awareness and limited access to health care^2^. In the absence of population-based studies, hospital-based studies would significantly underestimate the prevalence, severity, and patterns of UF in the population.

Symptomatic UF may be systematically different from nonsymptomatic UF. Studies show that there are marked differences in UF presentation, severity, treatment, and outcomes comparing Black and White women^2^. Black women typically develop UF at an earlier age, are more likely to have early-onset UF, and have larger, more numerous, and more rapidly growing UF^2,27,28^. These differences may be due to variations in genomic and other biological risk factors for UF^29–34^. Therefore, studies enrolling only clinically symptomatic UF are likely to misclassify cases, miss important risk factors, and lack the ability to detect variations in the impact of various risk factors on the etiopathogenesis, pathology, clinical presentation, progression, and outcomes of UF.

This is the first validation study of self-report of UF compared with gold standard TVUS in SSA. Our findings demonstrate the unreliability of self-report of either healthcare worker diagnosis or abdominal ultrasound diagnosis and a higher population prevalence of UF compared to the results obtained from hospital-based studies. Routine use of TVUS for evaluation and diagnosis is still relatively uncommon in SSA. Our findings should influence the design of future research on UF in SSA.

Despite the strengths of our study—it is population based, has a large sample size, and we were able to adjust for significant covariates—there are some limitations. We obtained TVUS only once during this study. UF may change in size or regress during the course of women’s reproductive lives. However, longitudinal studies suggest that spontaneous regression occurs in only a small proportion of cases^21,35–37^. Nevertheless, studies of spontaneous regressors may reveal additional insights into the aetiology and progression of UF, and collecting data about this phenomenon would be highly informative. We did not measure the number, size, or locations of the UF during TVUS in our study participants, nor did we obtain data on the histopathological characteristics of the UF. However, we believe that this information is unlikely to significantly change the results of this validation study. We used TVUS as the gold standard in this study, but we acknowledge that there are more sensitive methods for diagnosing and characterizing uterine fibroids, including magnetic resonance imaging (MRI), hysteroscopy, and sonohysterography. However, these methods are not practical for large-scale epidemiological research, particularly in LMICs^2,38–40^.

In conclusion, our study shows that self-report of UF is unreliable as a tool for epidemiological research on UF, and hospital-based studies significantly underestimate the prevalence of uterine fibroids in the population. Future studies of UF should use TVUS and similar tools and population-based study designs.

## Data Availability

The data will be made available upon request from the corresponding author.

## References

[1] Stewart EA, Cookson CL, Gandolfo RA, Schulze-Rath R (2017). Epidemiology of uterine fibroids: A systematic review. BJOG Int. J. Obstetr. Gynaecol..

[2] American College of Obstetricians and Gynecologists’ Committee on Practice Bulletins–Gynecology (2021). Management of Symptomatic Uterine Leiomyomas: ACOG Practice Bulletin, Number 228. Obstet. Gynecol..

[3] Baird DD, Dunson DB, Hill MC, Cousins D, Schectman JM (2003). High cumulative incidence of uterine leiomyoma in black and white women: Ultrasound evidence. Am. J. Obstet. Gynecol..

[4] Marshall LM, Spiegelman D, Barbieri RL (1997). Variation in the incidence of uterine leiomyoma among premenopausal women by age and race. Obstet. Gynecol..

[5] Wise LA, Palmer JR, Stewart EA, Rosenberg L (2005). Age-specific incidence rates for self-reported uterine leiomyomata in the Black Women's Health Study. Obstet. Gynecol..

[6] Agboola AD, Bello OO, Olayemi OO (2021). A clinical audit of the patterns of presentations and complications of abdominal myomectomy at the University College Hospital, Ibadan, Nigeria. J. Obstet. Gynaecol..

[7] Yorgancı A, Meydanlı MM, Kadıoğlu N (2020). Incidence and outcome of occult uterine sarcoma: A multi-centre study of 18604 operations performed for presumed uterine leiomyoma. J. Gynecol. Obstet. Hum. Reprod..

[8] Foth D, Rohl FW, Friedrich C (2017). Symptoms of uterine myomas: Data of an epidemiological study in Germany. Arch. Gynecol. Obstet..

[9] Soliman AM, Yang H, Du EX, Kelkar SS, Winkel C (2015). The direct and indirect costs of uterine fibroid tumors: A systematic review of the literature between 2000 and 2013. Am. J. Obstet. Gynecol..

[10] Morhason-Bello IO, Adebamowo CA (2022). Epidemiology of uterine fibroid in black African women: A systematic scoping review. BMJ Open.

[11] Mohammed A, Shehu S, Ahmed S (2005). Uterine leiomyomata: A five year clinicopathological review in Zaria, Nigeria. Niger. J. Surg. Res..

[12] Eze CU, Odumeru E, Ochie K, Nwadike U, Agwuna K (2013). Sonographic assessment of pregnancy co-existing with uterine leiomyoma in Owerri, Nigeria. Afr. Health Sci..

[13] Oluwole A, Owie E, Babah O, Afolabi B, Oye-Adeniran B (2015). Epidemiology of uterine leiomyomata at the lagos university teaching Hospital, Idi-Araba, Lagos. Niger. Hosp. Pract..

[14] Awowole IO, Makinde ON, Badejoko OO (2016). Clinical correlates of leiomyoma estrogen and progesterone receptors among Nigerian women. Int. J. Gynecol. Obstet..

[15] Sarkodie BD, Botwe BO, Adjei DN, Ofori E (2016). Factors associated with uterine fibroid in Ghanaian women undergoing pelvic scans with suspected uterine fibroid. Fertil. Res. Pract..

[16] Sarkodie BD, Botwe BO, Ofori EK (2016). Uterine fibroid characteristics and sonographic pattern among Ghanaian females undergoing pelvic ultrasound scan: A study at 3-major centres. BMC Womens Health.

[17] Egbe TO, Badjang TG, Tchounzou R, Egbe E-N, Ngowe MN (2018). Uterine fibroids in pregnancy: Prevalence, clinical presentation, associated factors and outcomes at the Limbe and Buea Regional Hospitals, Cameroon: A cross-sectional study. BMC Res. Notes.

[18] Wango EO, Tabifor HN, Muchiri LW, Sekadde-Kigondu C, Makawiti DW (2002). Progesterone, estradiol and their receptors in leiomyomata and the adjacent normal myometria of black Kenyan women. Afr. J. Health Sci..

[19] Tiltman AJ (1998). Leiomyomas of the uterine cervix: A study of frequency. Int. J. Gynecol. Pathol..

[20] Nigeria Population Commission (Nigeria), ICF (2019). Nigeria Demographic and Health Survey 2018: NPC.

[21] Myers SL, Baird DD, Olshan AF (2012). Self-report versus ultrasound measurement of uterine fibroid status. J. Womens Health.

[22] Adebamowo SN, Dareng EO, Famooto AO (2017). Cohort profile: African Collaborative Center for Microbiome and Genomics Research's (ACCME) Human Papillomavirus (HPV) and cervical cancer study. Int J Epidemiol.

[23] Muram D, Gillieson M, Walters JH (1980). Myomas of the uterus in pregnancy: Ultrasonographic follow-up. Am. J. Obstet. Gynecol..

[24] Gwet KL (2008). Computing inter-rater reliability and its variance in the presence of high agreement. Br. J. Math. Stat. Psychol..

[25] Landis JR, Koch GG (1977). The measurement of observer agreement for categorical data. Biometrics.

[26] Laberge PY, Vilos GA, Vilos AG, Janiszewski PM (2016). Burden of symptomatic uterine fibroids in Canadian women: A cohort study. Curr. Med. Res. Opin..

[27] Murji A, Bedaiwy M, Singh SS, Bougie O (2020). Influence of ethnicity on clinical presentation and quality of life in women with uterine fibroids: Results From a prospective observational registry. J. Obstet. Gynaecol. Can..

[28] Jacoby VL, Fujimoto VY, Giudice LC, Kuppermann M, Washington AE (2010). Racial and ethnic disparities in benign gynecologic conditions and associated surgeries. Am. J. Obstet. Gynecol..

[29] Al-Hendy A, Salama SA (2006). Ethnic distribution of estrogen receptor-α polymorphism is associated with a higher prevalence of uterine leiomyomas in black Americans. Fertil. Steril..

[30] Dvornyk V (2021). Significant interpopulation differentiation at candidate loci may underlie ethnic disparities in the prevalence of uterine fibroids. J. Genet..

[31] Wise LA, Ruiz-Narvaez EA, Palmer JR (2012). African ancestry and genetic risk for uterine leiomyomata. Am. J. Epidemiol..

[32] Piekos JA, Hellwege JN, Zhang Y (2022). Uterine fibroid polygenic risk score (PRS) associates and predicts risk for uterine fibroid. Hum. Genet..

[33] He C, Nelson W, Li H (2022). Frequency of MED12 mutation in relation to tumor and patient's clinical characteristics: A meta-analysis. Reprod. Sci..

[34] Bacanakgil BH, Ilhan G, Kaban I (2022). Variant type of leiomyomas: 13 years of experience in a single institution. Ginekol. Pol..

[35] Peddada SD, Laughlin SK, Miner K (2008). Growth of uterine leiomyomata among premenopausal black and white women. Proc. Natl. Acad. Sci. U.S.A..

[36] DeWaay DJ, Syrop CH, Nygaard IE, Davis WA, Van Voorhis BJ (2002). Natural history of uterine polyps and leiomyomata. Obstet. Gynecol..

[37] Ichimura T, Kawamura N, Ito F (1998). Correlation between the growth of uterine leiomyomata and estrogen and progesterone receptor content in needle biopsy specimens. Fertil. Steril..

[38] Munro MG, Critchley HOD, Fraser IS, Committee FMD (2018). The two FIGO systems for normal and abnormal uterine bleeding symptoms and classification of causes of abnormal uterine bleeding in the reproductive years: 2018 revisions. Int. J. Gynaecol. Obstet..

[39] Munro MG, Critchley HOD, Fraser IS (2019). Corrigendum to "The two FIGO systems for normal and abnormal uterine bleeding symptoms and classification of causes of abnormal uterine bleeding in the reproductive years: 2018 revisions". Int. J. Gynaecol. Obstetr..

[40] Obstetrics and Gynecology (2012). Diagnosis of abnormal uterine bleeding in reproductive-aged women: Practice Bulletin No. 128. Obstet. Gynecol..

